# Subdural Empyema Complicating Bacterial Meningitis: A Challenging Diagnosis in a Patient with Polysubstance Abuse

**DOI:** 10.1155/2015/931819

**Published:** 2015-10-12

**Authors:** Melissa Dakkak, William Russell Cullinane, Virin Rajiv Neil Ramoutar

**Affiliations:** Department of Medicine, University of Florida, 653 W. 8th Street, Box L18, Jacksonville, FL 32209, USA

## Abstract

Subdural empyema (SDE) and cerebrovascular accident (CVA) are uncommon life-threatening complications of bacterial meningitis, which require urgent neurosurgical intervention to prevent adverse outcomes. Clinicians must be vigilant of the onset of focal neurologic deficits or seizure activity to establish the diagnosis of SDE. *Streptococcus pneumoniae* accounts for <1% of pyogenic brain abscesses. This case describes a presentation of community acquired pneumococcal pneumonia in which the diagnosis of SDE with vasculitis induced CVA was confounded by concomitant substance abuse and sedation.

## 1. Introduction

*Streptococcus pneumoniae* is the most common cause of bacterial meningitis in adults but has been uncommonly reported as a cause of pyogenic brain abscess [1]. SDE is a less common complication of community acquired bacterial meningitis, seen in 2.7% of cases [2]. This sequela, although rare, must be considered in meningitis patients with concomitant otitis or sinusitis, focal neurologic deficits, epileptic seizures, and failure to improve clinically despite adequate antibiotics. However, the recognition of SDE may prove challenging in patients with altered sensorium from polysubstance abuse and ICU sedative medications as demonstrated in the case of this 44-year-old Human Immunodeficiency Virus (HIV) positive female.

## 2. Case Presentation

A 44-year-old Caucasian female with past medical history of schizoaffective disorder, HIV (CD4 of 499 cells/*μ*L), seizure disorder, and polysubstance abuse was brought into the emergency department (ED) after being found unresponsive in her room. It was reported that she ingested a bottle of pills of unknown identity. She had several similar presentations to hospital over the past 2 years. On initial assessment, her pupils were dilated but not fixed and she was unable to provide a history as she responded only to painful stimuli. Examination also revealed a supple neck, normal muscle tone throughout, and downgoing plantars. She was subsequently intubated for airway protection and transferred to the medical intensive care unit (ICU). Empiric acyclovir, vancomycin, and cefepime were given in the ED in the setting of leukocytosis to 16,300/mm^3^ and a low grade fever of 38.2 Celsius (100.8 Fahrenheit). Head computed tomography (CT) on admission revealed no acute ischemic or hemorrhagic stroke and chronic opacification of the right mastoid air cells and middle ear cavity (seen on CT images 8 months earlier). Infectious workup was performed and CSF culture was positive for* Streptococcus pneumoniae*. Urine drug screen was negative for benzodiazepines, cocaine, opiates, amphetamines, or barbiturates. Treatment doses of intravenous Ceftriaxone were started at 2 grams twice daily and continued based on antimicrobial sensitivities.

**Figure 1:**
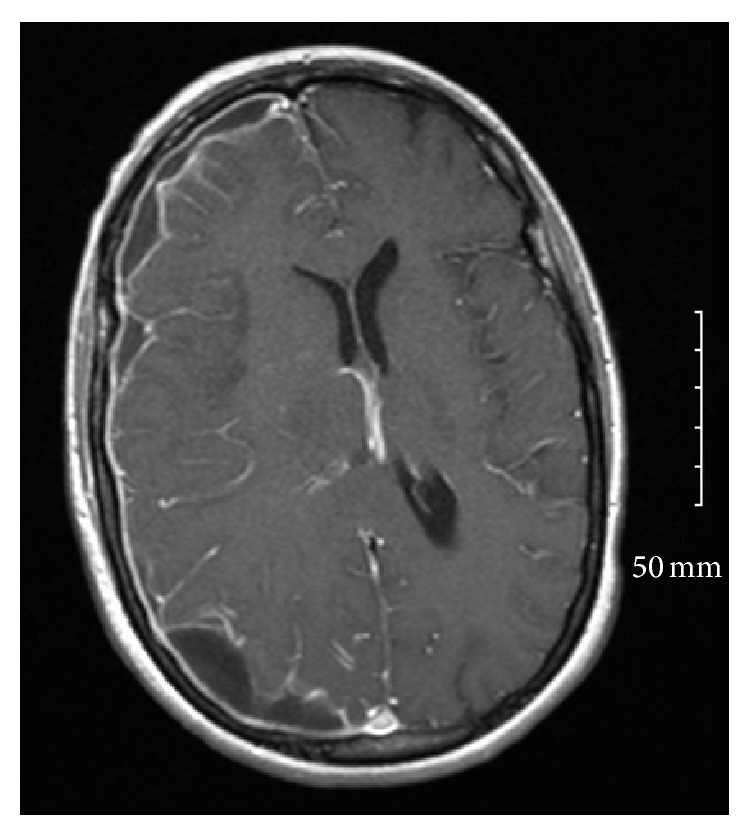
T1 MRI image demonstrating complicated right hemispheric subdural empyemas and right to left midline shift.

**Figure 2:**
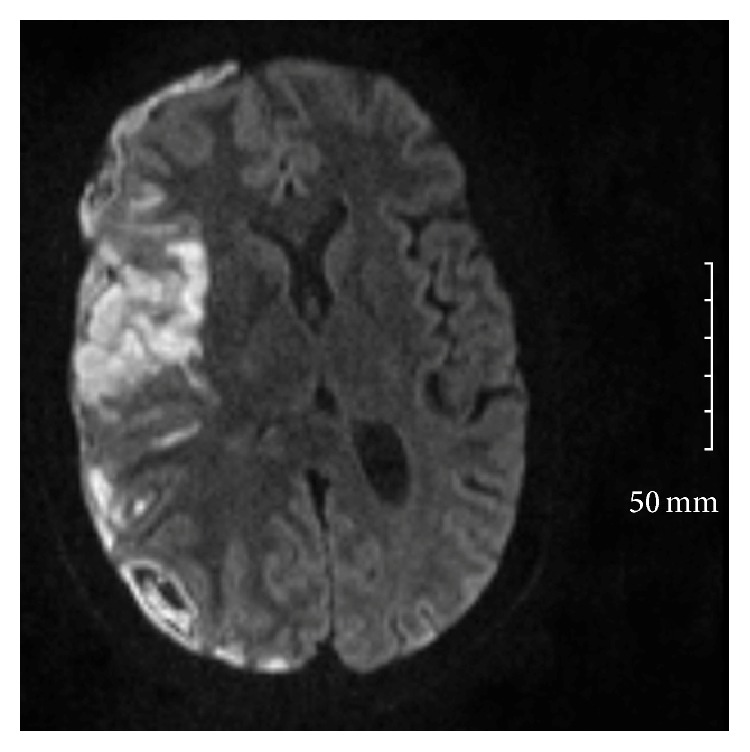
Diffusion Weighted Imaging (DWI) of the same MRI study demonstrating acute right middle cerebral artery territory infarct with loss of flow-related enhancement within the right middle cerebral artery. Per radiologist interpretation, findings may be secondary to associated vasculitis.

She was extubated and transferred to internal medicine service 4 days later with persistent altered mental state and agitation. This was attributed to residual effects of sedation used in ICU versus schizoaffective disorder and possible alcohol withdrawal. The patient was placed on haloperidol given her psychiatric history and lorazepam as needed for agitation or tremulousness secondary to alcohol withdrawal. The patient had intermittent fever spikes but normalizing white cell count (decreased from 16,300/mm^3^ to 10.1). However, after 24 hours the patient became less arousable despite not receiving additional sedative-hypnotics and was unable to protect her airway. After reintubation and transfer to medical ICU, she was noted to have preferential right gaze and left lower extremity weakness. A repeat head CT revealed possible venous temporal infarcts associated with adjacent subdural collection suggestive of an empyema. Follow-Up Magnetic Resonance Imaging (MRI) was obtained and is described in Figures 1 and 2.

The patient was transferred to the neurocritical care team and underwent a craniotomy for subdural empyema drainage 48 hours later owing to midline shift seen on MRI. A subdural drain was inserted intraoperatively and removed 3 days later, at which time the patient was successfully extubated. Follow-up MRI revealed right encephalomalacia with resolution of the subdural empyema 2 weeks after the drain was removed. A total of 4 weeks treatment with intravenous Ceftriaxone was completed. She had residual left upper extremity paresis with intact cognition upon discharge, when she was transferred to a Skilled Nursing Facility (SNF) for continued rehabilitation.

## 3. Discussion

Bacterial meningitis is a life-threatening disease that requires prompt medical attention. Pneumococci and meningococci are causative pathogens in approximately 80% of all cases with mortality from* S. pneumoniae* ranging from 19 to 37% [3]. The majority of patients present with two out of four of the following symptoms: headache, fever, neck stiffness, and altered mental status (as defined by a score of <14 on the Glasgow Coma Scale) [4]. This patient in this case presented with altered sensorium and low grade fever. However, given the history of pill ingestion and polysubstance abuse, the diagnosis of bacterial meningitis could easily have been missed.

In a review by Jim et al. [2], SDE was complication of 28 of 1,034 episodes (2.7%) of community acquired bacterial meningitis reported in a prospective Dutch cohort study from 2006 to 2011. 23 (82%) of these patients presented with neurologic symptoms of paresis, focal seizures, and dysthesia contralateral to the empyema. The patient described had a known history of seizure disorder and was given Divalproex at home doses, which may have explained the absence of seizures in this presentation. Further, a history of substance abuse and alcoholism favored the diagnosis of withdrawal and as needed sedative-hypnotics in the management of this patient. Failure to improve despite appropriate antibiotics and a decline in consciousness are the usual indicators that repeat brain imaging is warranted [3]. Rapid clinical deterioration and onset of seizures warrant the consideration of SDE as a complication of bacterial meningitis [5]. In the setting of sedative-hypnotic medication, intubation, psychiatric illness, and possible withdrawal repeat imaging can be easily overlooked.

In the Dutch study [2], 21 (75%) patients had concomitant otitis or sinusitis with contiguous spread to the subdural space. In this case, there was the opacification of the mastoid air cells and right middle ear cavity that was suggestive of an effusion or infectious process. The patient was unable to provide a history on presentation and prior images revealed similar findings so the diagnosis of otomastoiditis was not initially considered. An immunocompromised state was also seen in 8 (29%) of the 28 reported cases of SDE in the Dutch cohort. The incidence of SDE in pneumococcal meningitis patients presenting with otitis was high at 8% [2]. Historically,* S. pneumoniae* was thought to be a rare cause of pyogenic brain abscess occurring in less than 1% of all reported cases [1, 6, 7]. However, pneumococcus was identified in 26 (93%) of the 28 patients with SDE in the Dutch cohort [2].

Vascular complications are common in bacterial meningitis, occurring in 15–20% of all infections and in as many as one-third of patients with pneumococcal meningitis. Small vessel vasculitis and vasospasm are the primary mechanisms implicated [8] and this is well demonstrated in Figure 2. Cerebral infarcts may involve large vascular territories with subsequent brain edema and mass effect leading to a decline in consciousness [4]. In this case, the patient suffered a right middle cerebral artery (MCA) territory infarction and appropriately underwent neurosurgical intervention given the presence of midline shift and SDE. Neurosurgical intervention should be regarded as the first-line therapy in patients with SDE causing midline shift and focal neurologic abnormalities or a decreased level of consciousness [2]. The optimal duration of antimicrobial therapy has not been established in trials but generally 3- to 4-week period is advised if an empyema has been evacuated and even longer if conservatively managed [9]. In this case, the patient completed 4 weeks of Ceftriaxone intravenously in keeping with antimicrobial sensitivities.

## 4. Conclusion

The diagnosis of bacterial meningitis in itself can be challenging with a low sensitivity for the classic triad of fever, neck stiffness, and altered mental state. When the less common complication of SDE is superimposed on the hospital course, this represents an even greater challenge to physicians. In cases where clinical presentation is confounded by polysubstance abuse and the need for sedation in the setting of intubation, vigilance must be increased for complications of meningitis. It is suggested that there be a low threshold for repeat brain imaging when initial films reveal evidence of opacification in the sinuses, ear cavity, or adjacent structures. Further, the goal in the patient with meningitis should always be to minimize sedation to ensure that neurological status can be properly assessed. Prompt neurosurgical intervention is warranted when SDE leads to midline shift, focal neurologic abnormalities, or decreased level of consciousness.
