# Corrigendum to “Subdural Empyema Complicating Bacterial Meningitis: A Challenging Diagnosis in a Patient with Polysubstance Abuse”

**DOI:** 10.1155/2017/6145716

**Published:** 2017-11-14

**Authors:** Melissa Dakkak, William Russell Cullinane, Virin Rajiv Neil Ramoutar

**Affiliations:** Department of Medicine, University of Florida, 653 W. 8th Street, Box L18, Jacksonville, FL 32209, USA

In the article titled “Subdural Empyema Complicating Bacterial Meningitis: A Challenging Diagnosis in a Patient with Polysubstance Abuse” [1], there was an error in the abstract section, where “pneumococcal pneumonia” should be corrected to “pneumococcal meningitis.”
